# Monitoring Single-Phase LV Charging of Electric Vehicles

**DOI:** 10.3390/s23010141

**Published:** 2022-12-23

**Authors:** Piotr Kuwałek, Grzegorz Wiczyński

**Affiliations:** Institute of Electrical Engineering and Electronics, Poznan University of Technology, Piotrowo Street, No. 3a, 60-965 Poznan, Poland

**Keywords:** charging process, electric vehicles, power quality monitoring, reliability of electrical machines

## Abstract

The paper presents the results of the monitoring process of the charging of an electric vehicle battery pack. Battery pack charging with a capacity of 58kWh was monitored in a single-phase 230V/50Hz circuit. The slow charging system used was configured to obtain a current of 10A. During monitoring, the focus was on the recognition of the charging, considering the impact of this process on power quality and, consequently, on the reliability of electrical machines. Research results show that the monitored charges are one-, two-, or three-stage processes. The variations in the currents, power, and higher harmonic contents were observed. The effects of such variations depend on the properties of the power grid at the point of connection of the charging system. Knowledge of the variation of the voltages, currents, and active and reactive power allows for the determination of the requirements of the measuring equipment used for charging the monitoring, including the selection of discrimination/averaging time of monitored quantities. The research results also indicate the need for continuous monitoring of the power quality in the power supply circuit of electrical loads, e.g., electrical machines. Continuous monitoring supports the diagnostics of electrical machines and allows the appropriate measures to increase their reliability.

## 1. Introduction

In the power grid, multiple disturbances of power quality are common, such as voltage distortion caused by higher harmonics, voltage asymmetry, and voltage fluctuations [1]. Disturbances in the power quality have negative impacts on particular elements of the power system, including electric machines [2,3], causing, e.g., their overheating [4], excessive vibrations [5], torsional vibrations [6], reduced efficiency [7], loss of reliability, and durability [8,9,10]. Power quality disturbances that occur in the power grid and affect the reliability of electrical machines include, among others, voltage fluctuations [11,12], voltage distortions caused by higher harmonics [13] or inter-harmonics [14,15]. At the same time, in some cases, the operation of electric machines or devices associated with them can also cause disturbances of power quality [16,17,18], for example, the electric machine control or the charging of an electric vehicle (EV) [19,20]. The consequence of the appearance of the reduced power quality is the reduction of the reliability of other loads (including electric machines) supplied from the same power grid. Therefore, it is necessary to continuously monitor the parameters determining the power quality [21] to identify early the occurrence of disturbances of power quality and take appropriate actions (e.g., activation of active parallel filters [22,23,24], activation of the voltage stabilizers [25,26], and activation of intelligent correction systems of the quality level of the supply voltage [27,28]) to reduce the potential effects of disturbances of power quality. Therefore, monitoring the parameters of the supply voltage [29] for individual power loads (including electrical machines) contributes to increasing their reliability.

In recent years, there has been a significant increase in interest in electric vehicles. In response to this trend, appropriate actions are taken to adapt the power infrastructure (e.g., fast charging stations for electric vehicles are installed in power grids). Electric vehicles can also be charged in households using appropriately adapted single-phase chargers. This method of the charging has become popular in recent years due to the convenience of use and economic aspects [30]. At the same time, this method of the charging is the worst from the point of view of power quality, due to the low short-circuit power in low-voltage power grids, especially in the cases of the charging of electric vehicles in households located at a considerable distance from the power station (consumers at the end of the line in the radial topology of low-voltage (LV) networks). In such situations, there are increased phenomena that reduce the power quality and, as a consequence, have negative impacts on other power loads, which are supplied from the same power grids where electric vehicles are charged. In recent years, many problems caused by the charging of electric vehicles have been identified, e.g., voltage distortion caused by higher harmonics [31,32,33,34], voltage variation caused by the dynamics of electric vehicle charging processes [35,36,37,38], and the generation of supraharmonics caused by the power electronics of electric vehicle chargers [39,40]. It is worth noting that in the indicated research results, an attempt is made to generalize, joining certain types of electric vehicle chargers with specific power quality disturbances, which are strictly defined in their nature. On this basis, many methods of evaluation and counteraction of interference caused by electric vehicle chargers are defined [41,42,43,44]. However, in the real power grid, there is a mutual interaction between the current forced by the electric vehicle chargers, which affects the process of the charging of batteries of electric vehicles and the quality of the supply voltage. Such a state may hinder the actions aimed at minimizing disturbances caused by electric vehicle chargers. Therefore, it is necessary to continuously study and monitor the variations of processes during the charging of electric vehicles, so that solutions that improve power quality are effective [45,46].

The paper presents the selected results of monitoring the charging process of an electric vehicle from the single-phase low-voltage (LV) network. During the monitoring, the non-stationarity of the charging process is observed, within which it is possible to indicate a different number of stages, which (to a certain extent and in different ways) affect the reduction of power quality. The long-term results of the observations indicate that even for one specific type of EV onboard charger supplied from the same supply circuit (the supply circuit with the high short-circuit power in the LV network), different natures of EV charger processes are observed each time, characterized by different changes in parameters determining the power quality. The presented research results indicate the need for continuous monitoring of the quality of the supply voltage for electric machines, especially when the electric vehicle is charged in the same supply circuit because the effects of the impacts during vehicle charging can be difficult to predict due to the non-stationarity of this process. Unusual cases are also presented, in which the relationship between the individual harmonics of the EV charger current and its supply voltage is notable (the strong potential impact of the EV charger on the supply voltage despite the low impedance of the supply circuit). Section 2 presents the research object and discusses research conditions. Section 3 presents and discusses the obtained measurement results. Section 4 presents the final conclusions resulting from the presented selected results of the research carried out.

## 2. Materials and Methods

**Figure 1:**
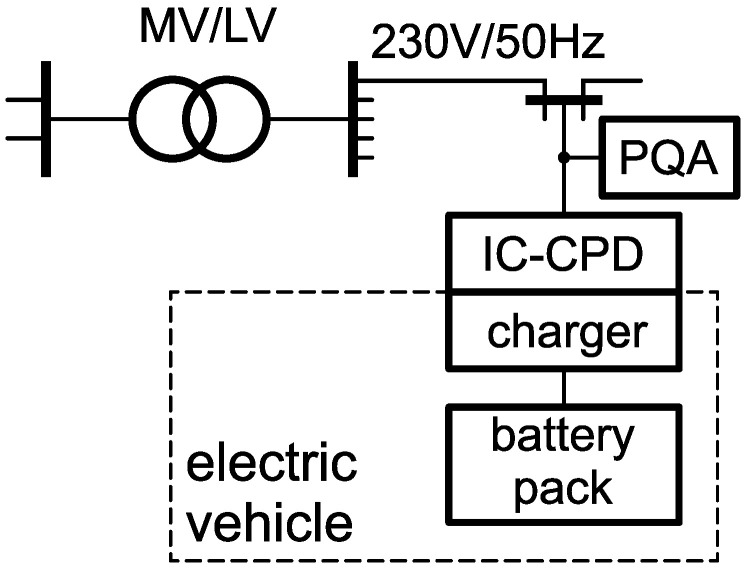
The diagram of the battery pack charging circuit with the measurement point marked, where MV/LV is the medium-voltage to low-voltage transformer, PQA is the power quality analyzer, IC–CPD is the in-cable control and protection device, and the charger is the battery pack charging process control system.

The research object involved charging the battery pack of an electric vehicle from the side of the power grid. The charging process was monitored in a three-phase 230V/50Hz low-voltage (LV) network. The public buildings were also supplied from the same supply circuit to which the charger was connected. There were different loads in public buildings that affected the supply voltage. The battery pack was charged in a single-phase circuit. The length of the power line (from the MV/LV transformer to the supply point of the charging system, where MV is the medium voltage) is short. Thus, the impedance of the power supply circuit was low, and the impact of the charging system on the power grid was negligible. The diagram of the power supply circuit of the tested battery pack charging system is shown in Figure 1.

**Figure 2:**
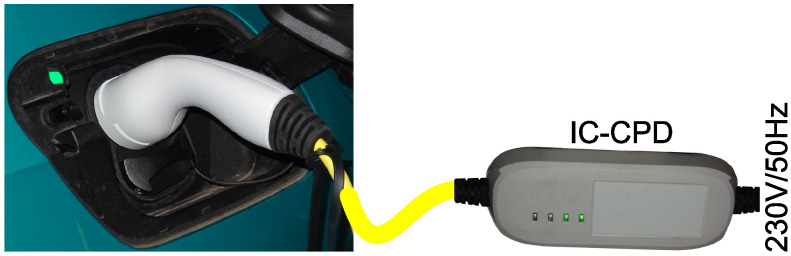
The photo of the connector in electric vehicle (during charging) and the charging current controller (IC–CPD).

The battery pack is a set of 216 lithium-ion cells with a nominal voltage of 3.65V and a capacity of 78Ah. The total capacity of the pack is 62kWh with a usable capacity of 58 kWh (94%). The IC–CPD system with a 230V/50Hz power grid is configured to consume 10A of the current (mode 2 [47]). During charging, the IC–CPD system was supplied with a non-stabilized voltage (changed according to the voltage on the MV side and the power consumption of loads, which are supplied from the same supply circuit). There is no detailed information about the charger. Despite attempts to obtain technical documentation for the tested charger, the manufacturer refused to provide detailed technical information, citing the confidential data clause. Figure 2 shows a photo of an electric vehicle whose charging process was monitored.

The process of charging an electric vehicle, as indicated in Figure 2, was monitored from May to November 2022. During the monitoring, parameters determining power quality [48] were recorded: fundamental voltage frequency, rms value of the voltage *U* and current *I*, active *P* and reactive *Q* power, active $E_{P}$ and reactive $E_{Q}$ energy, higher harmonics of the voltage $u_{h}$ and current $i_{h}$, total harmonic distortion (THD) factor of the voltage THDU and current THDI, short-term $P_{st}$ and long-term $P_{lt}$ flicker indicator, asymmetry coefficients, voltage fluctuation indices, with the use of the SPE energy power quality analyzer [49]. The indicated parameters (maximum, average, and minimum values) were recorded in intervals from 10s to 60s, except for the indicator $P_{st}$, which was recorded with an interval of 10min. The measurement uncertainty of:

- rms value of the voltage *U*: ±0.1%$U_{\mathrm{din}}$, where the declared input voltage $U_{\mathrm{din}}$ = 230V;
- indicator $P_{st}$: 0.05 for $P_{st}\leq 1$ and 5%$P_{\mathrm{stm}}$ for $P_{st}>1$, where $P_{\mathrm{stm}}$ is the measurement result;
- individual harmonics: 0.05%$U_{\mathrm{din}}$ for $U_{h}$ <0.01$U_{\mathrm{din}}$ and 5%$U_{h}$ for $U_{h}\geq 0.01U_{din}$;
- Total Harmonic Distortion (THD) factor of the voltage THDU and current THDI: 0.05%;
- rms value of the current *I*: ±0.1%$I_{\mathrm{range}}$, where the current range $I_{\mathrm{range}}$ = 30A;
- active power: $\pm 0.15\%\left(U_{\mathrm{din}}\cdot I_{\mathrm{range}}\right)$;
- reactive power (according to Budeanu’s concept): $\pm 0.2\%\left(U_{\mathrm{din}}\cdot I_{\mathrm{range}}\right)$.

Phase voltages were measured directly. The current clamp Kyoritsu KEW8146 [50] was used to measure the EV charger current (rated current: 30A; output voltage: 50mV/A; measurement uncertainty in the range 0–15A: ±1.0% of reading (50/60Hz) and ±2.0% of reading (40Hz–1kHz); dimensions: 100 × 60 × 26mm; max. conductor diameter: 24mm). The measurement inaccuracies of parameters determining the power quality by the used power quality analyzer were at the levels specified by the standard IEC 61000-4-30 [51]. The monitoring of the indicated parameters was carried out during the charging of the electric vehicle, as well as when the electric vehicle was not being charged, to know the nature of power loads, which were supplied from the same supply circuit as the electric vehicle charger. Such actions carried out in the long-term make it possible to minimize the probability of incorrect conclusions about the impact of the EV onboard charger on the power quality. Each time, the EV charging process ended with 80% of the battery charge according to the algorithm set in the EV onboard charger control system. During measurements, information about changes in the actual state of the charge of the battery was not recorded.

The paper presents five selected examples of battery pack charging for different discharge states. Table 1 summarizes information on active and reactive energy supplied during the charging process. Selected examples are characterized by different numbers of stages with different numbers of sub-stages that were observed during the charging process. The *X*-th stage (stage Xi) is understood as a state in which the nature of capacitive reactive power consumed by the system is quasi-stationary. Thus, a step change regarding the reactive power consumed results in the separation of the next stage. In turn, a step change in the nature of the active power variation associated with a step change in at least one other selected recorded parameter results in the separation of the *i*-th sub-stage of the *X*-th stage (stage Xi). The other recorded parameters were, e.g., the minimum $P_{\min}$, average $P_{\mathrm{avg}}$, and maximum $P_{\max}$ values of active power, the THDU factor of supply voltage, the THDI factor of consumed current, and individual *h*-th higher harmonics of the current $i_{h}$, and voltage $u_{h}$.

The battery pack charging process is performed in the constant current (CC) or the constant voltage (CV) mode [52,53,54]. The charging process starts in CC mode. For this mode of the charging process, a slow increase in the charging power can occur due to a slow increase of the voltage on the battery pack and it cannot cause short-term changes in the current and power of the EV onboard charger. Moreover, the CV mode does not cause rapid changes in power consumption. It should be noted that such EV onboard chargers are power electronic systems with built-in regulators [55,56]. For example, a power factor compensation (PFC) [54,57] is commonly built in. Such a system includes a regulator whose task is to obtain a power factor cosφ with a value close to 1. The operation of regulators, for example, in interaction with changes in the supply voltage [57,58,59], can cause additional changes in the power consumed by EV onboard chargers, in proportion to the control algorithm used.

## 3. Results and Discussion

### 3.1. Case I

**Figure 3:**
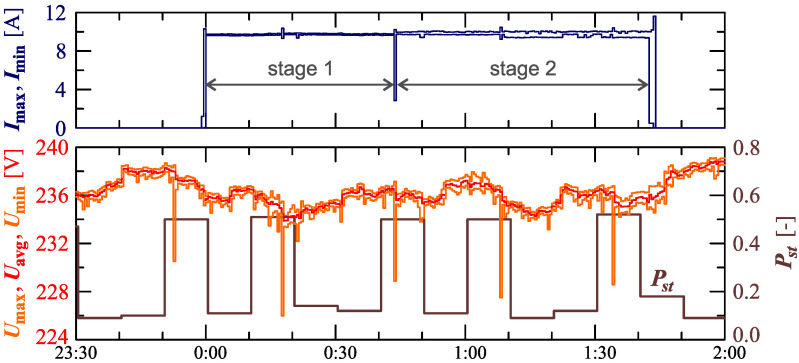
The characteristics of maximum $U_{\max}=f\left(t\right)$, average $U_{\mathrm{avg}}=f\left(t\right)$ and minimum $U_{\min}=f\left(t\right)$ values of rms values of supply voltage and characteristic $P_{st}=f\left(t\right)$ for Case I.

The charging system was supplied with non-stabilized voltage from the LV power grid during the monitoring, as mentioned in Section 2. In the monitoring process, the supply voltage values are changing, as shown in Figure 3. The variations of RMS values of the voltages are shown in the characteristics $U_{\max}=f\left(t\right)$, $U_{\mathrm{avg}}=f\left(t\right)$ and $U_{\min}=f\left(t\right)$ (where: $U_{\max}$, $U_{\mathrm{avg}}$ and $U_{\min}$ are, respectively, the maximum, average, and minimum rms values determined for the interval $T_{\mathrm{REC}}$ = 30s) along with the characteristic $P_{st}=f\left(t\right)$. Figure 3 also shows the characteristics of maximum $I_{\max}=f\left(t\right)$ and minimum $I_{\min}=f\left(t\right)$ values of rms values of the current.

Figure 3 shows slow voltage changes (in the range of ±6V) with short-term decreases every 27 min. By monitoring voltage variations in a weekly time horizon (during charging and beyond), it was found that short-term voltage decreases are caused by the cyclical operation of a disturbing load active for some part of the day. It should be emphasized that rms values of the voltages meet the normative requirements of standard EN 50160 [48], i.e., rms values of the voltages are in the range of 230V± 10%. Moreover, voltage fluctuations, expressed by the short-term flicker indicator $P_{st}$, meet the normative requirements of the standard EN 50160 [48] ($P_{st}\leq 1$).

**Figure 4:**
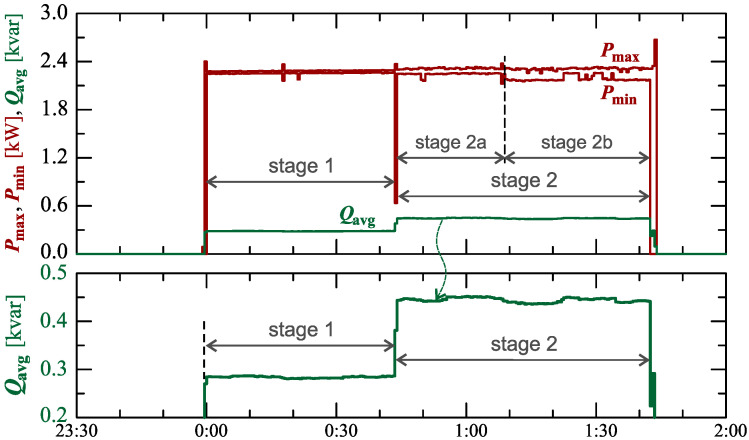
The characteristics of maximum $P_{\max}=f\left(t\right)$ and minimum $P_{\min}=f\left(t\right)$ values of active power and average values of reactive power $Q_{\mathrm{avg}}=f\left(t\right)$ for Case I.

Figure 4 shows the maximum $P_{\max}$ and minimum $P_{\min}$ characteristics of active power and average reactive power $Q_{\mathrm{avg}}$ during active charging for 104min.

Regarding the characteristics of reactive power (Figure 4), two stages can be easily indicated. In addition, there are 2 sub-stages in stage 2 (stage 2a and stage 2b).

**Figure 5:**
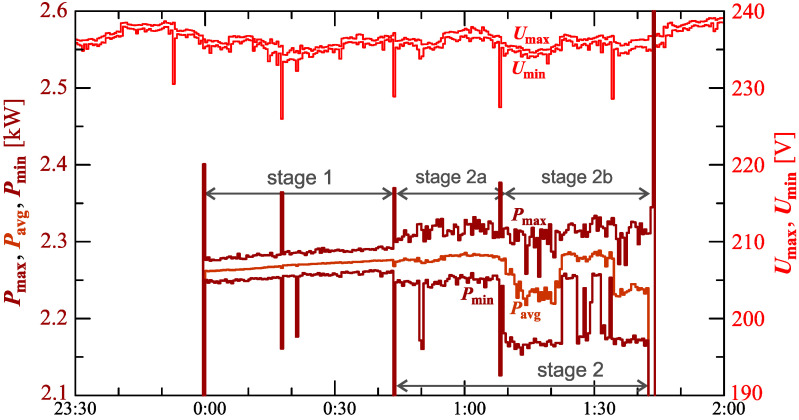
The characteristics of maximum $P_{\max}=f\left(t\right)$, average $P_{\mathrm{avg}}=f\left(t\right)$, and minimum $P_{\min}=f\left(t\right)$ values of active power and maximum $U_{\max}=f\left(t\right)$ and minimum $U_{\min}=f\left(t\right)$ rms values of the supply voltage for Case I.

Figure 5 shows the characteristics of maximum $P_{\max}$, average $P_{\mathrm{avg}}$ and minimum $P_{\min}$ values of active power (on a properly scaled characteristic).

The characteristics of Figure 5 in stage I show a slow linear increase in the active power consumed. This can indicate that the battery pack is charging in a constant current mode. In stage 2 (at stage 2a and stage 2b), the charging mode changes while the increases in active power change. Short-term undervoltages cause short-term increases in active power changes. However, due to the short duration of this phenomenon, it does not have a significant impact on the charging process.

**Figure 6:**
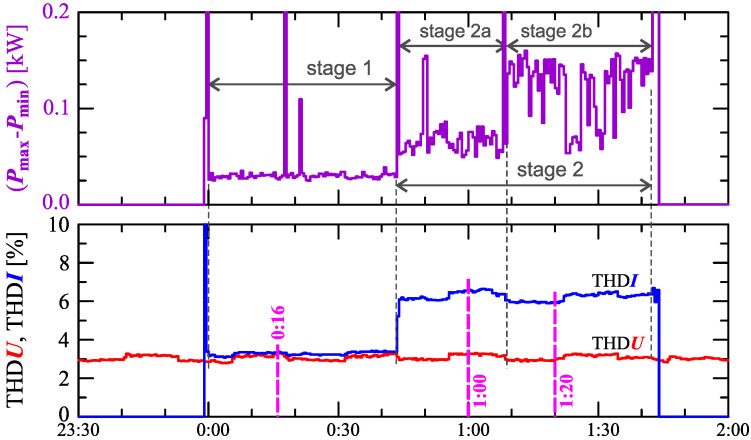
The differences between the maximum and minimum active power $\left(P_{\max}-P_{\min}\right)=f\left(t\right)$ (top), and the voltage THD factor THDU=f(t) and current THD factor THDI=f(t) (bottom) with the marking of measurement results presented on the waveforms (Figure 11) and amplitude spectra (Figure 10) recorded at 0:16, 1:00, and 1:20 for Case I.

Figure 6 shows the difference between the maximum $P_{\max}$ and minimum $P_{\min}$ active power determined for the interval $T_{\mathrm{REC}}$ = 30s.

The characteristic in Figure 6 clearly shows the increase in active power changes $\left(P_{\max}-P_{\min}\right)$ in successive stages. In stage 1, the variation is approximately 30W, in stage 2b, it increases to approximately 150W.

The indicated stages of the charging process are determined by analyzing the nature of the active and reactive power. However, stages/sub-stages of the charging process can be determined by analyzing the current and voltage harmonics. Figure 6 also shows the characteristics of the current and voltage THD factors. Comparing the characteristics of the current and voltage THD factors, it can be noticed that there is no dependency between the changes in the current and voltage distortions (i.e., the increase in the current distortion is not caused by the increase in the voltage distortion). The current THD factor suggests the division of the charging process into two stages. In stage 1, the nature of the current and voltage THD factors are similar. In stage 2, the values of the current THD factor increase while the values of the voltage THD factor is kept (approximately) constant.

**Figure 7:**
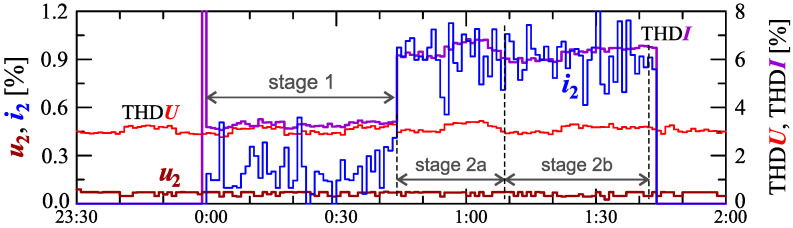
The characteristics of the voltage THD factor THDU=f(t) and the current THD factor THDI=f(t) and the characteristics of the second harmonics of the current $i_{2}=f\left(t\right)$ and voltage $u_{2}=f\left(t\right)$ for Case I.

Information about the charging process can be provided by analyzing individual higher harmonics. Considering the properties of discrete Fourier transform (DFT), even harmonics can be expected in the case of power variation (thus, current variations). Figure 7 shows characteristics of the voltage and current THD factors and the characteristics of the second harmonics of the current and voltage.

As expected, the occurrence of the second harmonic of the current is correlated with the occurrence of the current variation. The characteristic of this harmonic, similar to the characteristic of the current THD factor, suggests the division of the charging process into two stages. Information about the charging process can be obtained by analyzing higher harmonics.

**Figure 8:**
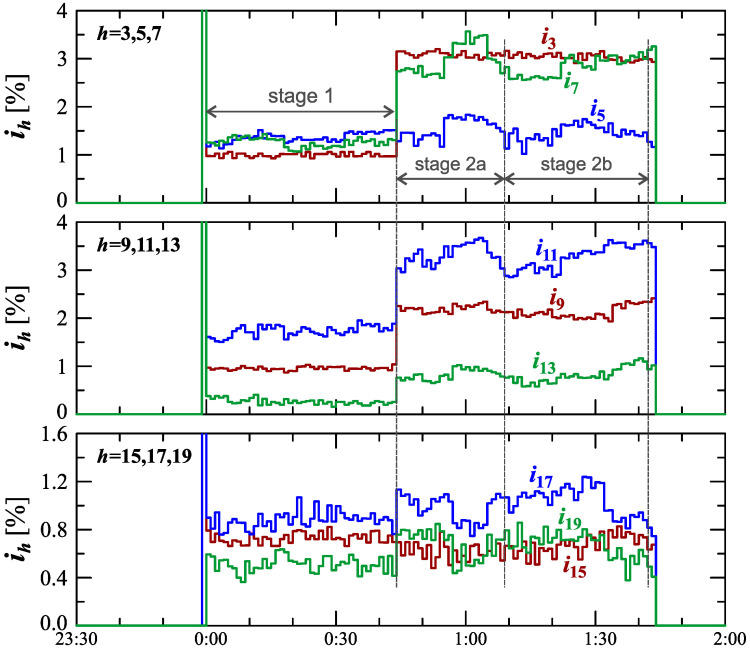
The characteristics of odd harmonics of the current $i_{h}=f\left(t\right)$ from the order *h* = 3 to 19 for Case I.

**Figure 9:**
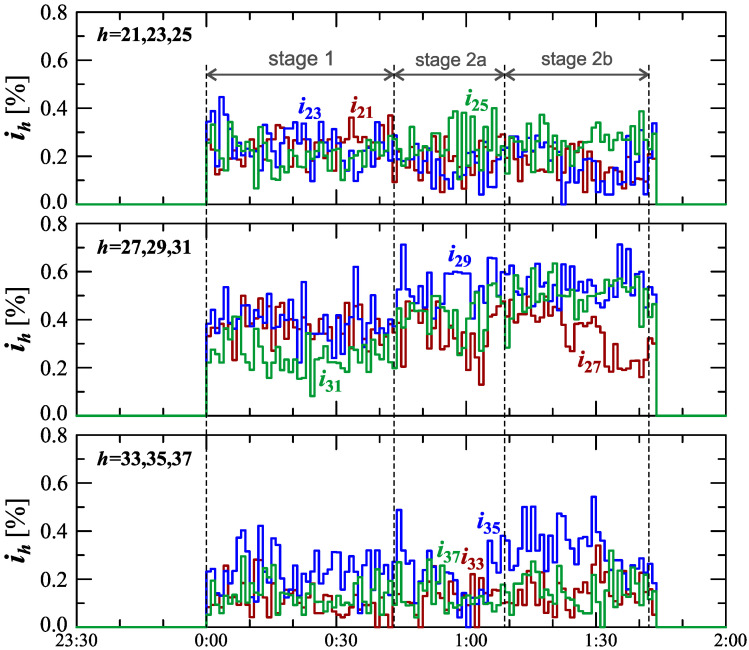
The characteristics of odd harmonics of the current $i_{h}=f\left(t\right)$ from the order *h* = 21 to 37 for Case I.

The characteristics of odd harmonics of the current $i_{h}$ from the order *h* = 3 to 19 are shown in Figure 8. Analyzing Figure 8, an analogous trend for the 2nd order harmonic can be seen, similar to the odd harmonics from the 3rd to the 13th order when charging an electric vehicle. For the 5th harmonic, there is the same strong correlation with the current THD factor as for the 2nd harmonic. In stage 1, after the start of the charging, the individual harmonics first increase to a quasi-determined level. In stage 2, during vehicle charging, when the power variation increases, subsequent increases in the values of individual harmonics are observed. Moreover, individual sub-stages (stage 2a and stage 2b) can be observed in the case of characteristics of the 5th-, 7th, 11th-, and 13th-order harmonics. The indicated harmonics in stage 2 are periodically monotonic, one sub-stage (stage 2a/stage 2b) has a period of increase and a period of decrease in the value of individual harmonics. The indicated harmonics in stage 2 are probably related to the increase in the impact of the power electronic converter in the charging system, which is associated with the harmonics of the order of 6n±1, where *n* is any natural number. During charging an electric vehicle, there are also harmonics in the current higher orders than harmonics presented in Figure 8. This can be seen in Figure 9. Analyzing Figure 9, it can be seen that during the charging of an electric vehicle, the contribution of higher harmonics from the order of 15 is also revealed. In the case of these harmonics, there is no visible change in their nature in individual stages and sub-stages. During the entire charging process, harmonics higher than the 15th order fluctuate around one quasi–definite level, the value of which is different for individual harmonics. The occurrence of higher harmonics indicates that the signal is distorted.

**Figure 10:**
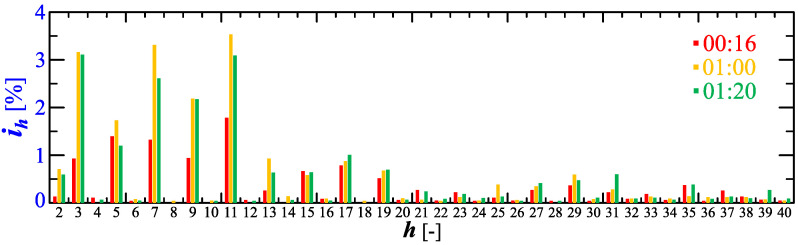
The amplitude spectrum of the current $i_{h}=f\left(h\right)$ recorded at 00:16, 1:00, and 1:20 for Case I.

**Figure 11:**
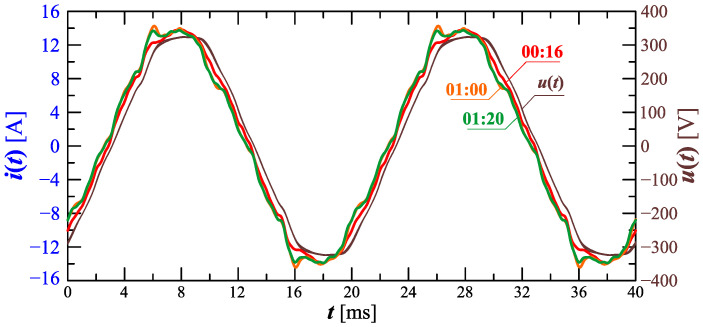
Recreated voltage waveforms u(t) and current waveforms i(t) recorded at 00:16, 1:00, and 1:20 for Case I.

Figure 10 shows the amplitude spectrum of the current recorded at 00:16, 1:00, and 1:20 (recording times are marked in Figure 6). Recreated current waveforms on the basis of recorded harmonics are shown in Figure 11.

**Figure 12:**
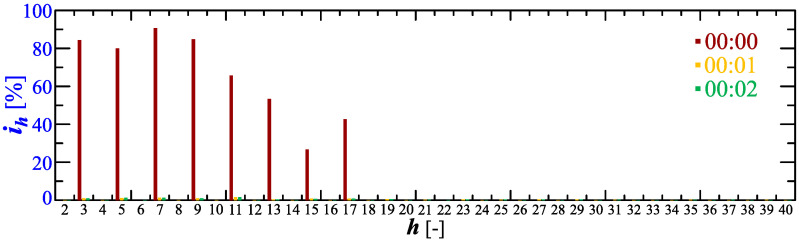
The amplitude spectrum of the current $i_{h}=f\left(h\right)$ recorded at 00:00, 00:01, and 00:02 for Case I.

**Figure 13:**
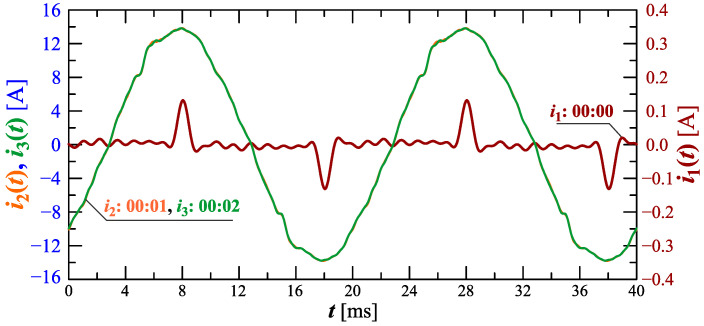
Recreated voltage waveforms u(t) and current waveforms i(t) recorded at 00:00, 00:01, and 00:02 for Case I.

Analyzing characteristics of the current THD factor in Figure 6 and Figure 7, it can be seen that in the initial charging phase (approximately 00:00) the deformation increases. The start of the charging is depicted in Figure 12 and Figure 13 showing the amplitude spectrum and current waveforms at 00:00, 00:01, and 00:02.

Analyzing characteristics from Figure 10, Figure 11, Figure 12 and Figure 13, one can notice a significant non-stationarity of the charging process, characterized by current waveform changes, and, thus, the changes in the contribution of individual harmonic values, in subsequent time steps. Based on the waveforms presented in Figure 11 and Figure 13, conclusions can also be made regarding the requirements of measurement equipment used to measure selected quantities during the charging of an electric vehicle. Namely, it is worth noting that the current waveform is strongly non-stationary in time, and as a consequence, it can lead to significant errors in the measurement of higher harmonics (e.g., by carrying out the measurement in accordance with the requirements of standard IEC 61000-4-30 [51] referring to the standard IEC 61000-4-7 [60] for harmonics measurement) due to the phenomenon of “spectrum leakage” [61]. In such situations, considering quasi-periodic states (such as those presented in Figure 11 and Figure 13), it is necessary to detect fundamental periods using advanced signal processing methods (e.g., using the ESPRIT method [62]). Only after the effective detection of indicated quasi-fundamental periods is it possible to correctly determine all parameters calculated for the periodic waveforms (rms values of higher harmonics, THD factor, rms value of signal, active and reactive power). It is also worth noting the shapes of the separated quasi-periods of the current waveforms in time. For this type of waveform, the value of the crest factor is much more different from $\sqrt{2}$ (see Figure 13), so the use of equipment based on a crest detector also leads to significant errors in the measurement of the rms value of the current during the charging of an electric vehicle. On the other hand, in the case of the measurement equipment that allows the true rms to be determined, the aspect of the appropriate selection of the fundamental period occurs.

### 3.2. Case II

**Figure 14:**
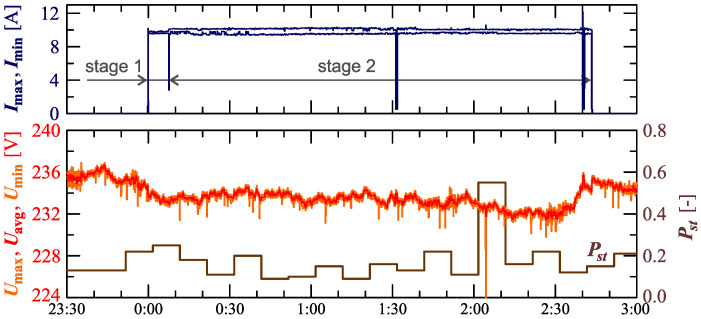
The characteristics of maximum $U_{\max}=f\left(t\right)$, average $U_{\mathrm{avg}}=f\left(t\right)$, and minimum $U_{\min}=f\left(t\right)$ values of rms values of the supply voltage and characteristic $P_{st}=f\left(t\right)$ for Case II.

During the charging process defined as Case II, as in Case I, the supply voltage changes, as shown in Figure 14. The variation of rms voltage values is shown in the characteristics $U_{\max}=f\left(t\right)$, $U_{\mathrm{avg}}=f\left(t\right)$ and $U_{\min}=f\left(t\right)$ (where: $U_{\max}$, $U_{\mathrm{avg}}$ and $U_{\min}$ are respectively the maximum, average, and minimum rms values determined for the interval $T_{\mathrm{REC}}$ = 10s) along with the characteristic $P_{st}=f\left(t\right)$. Figure 14 also shows the characteristics of maximum $I_{\max}=f\left(t\right)$ and minimum $I_{\min}=f\left(t\right)$ values of rms values of the current.

Figure 14 shows slow voltage changes (typically in the ±4V range) with short-term decreases every few minutes. As in Case I, the observed voltage variation in a weekly time horizon (during charging and beyond) indicates that short-term undervoltages are caused by the cyclical operation of a disturbing load active for part of the day, unrelated to the tested vehicle charging system. It should be emphasized that the rms values of the voltage are in the range of 230V± 10%. Voltage fluctuations expressed by the short-term flicker indicator $P_{st}$, as in Case I, meet the normative requirements of standard EN 50160 [48] ($P_{st}\leq 1$).

**Figure 15:**
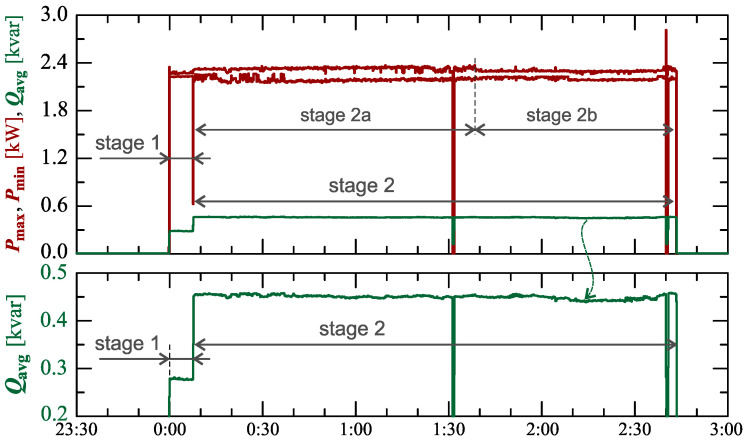
The characteristics of the maximum $P_{\max}=f\left(t\right)$ and minimum $P_{\min}=f\left(t\right)$ values of active power and average values of reactive power $Q_{\mathrm{avg}}=f\left(t\right)$ for Case II.

Figure 15 shows the characteristics of the maximum $P_{\max}$ and minimum $P_{\min}$ of the active power and average reactive power $Q_{\mathrm{avg}}$ during the active charging for 164min. Analyzing the characteristics of the reactive power (Figure 15), two stages can be identified, but in stage 2, two sub-stages can be separated (stage 2a and stage 2b).

**Figure 16:**
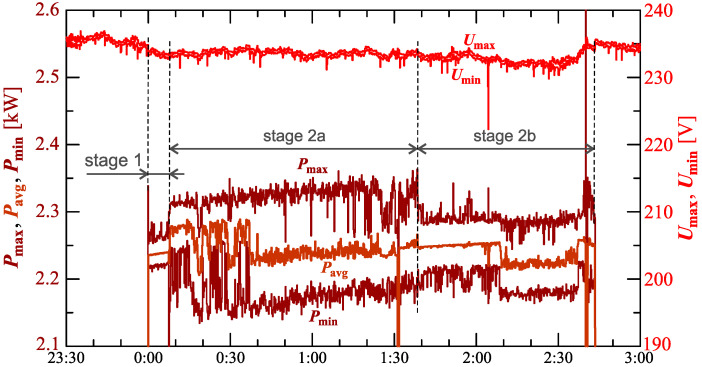
The characteristic of the maximum $P_{\max}=f\left(t\right)$, average $P_{\mathrm{avg}}=f\left(t\right)$, and minimum $P_{\min}=f\left(t\right)$ values of the active power and maximum $U_{\max}=f\left(t\right)$ and minimum $U_{\min}=f\left(t\right)$ rms values of the supply voltage for Case II.

Figure 16 shows the characteristics of the maximum $P_{\max}$, average $P_{\mathrm{avg}}$, and minimum $P_{\min}$ of the active power, on a properly scaled characteristic, in relation to the characteristics of maximum $U_{\max}$ and minimum $U_{\min}$ rms values of the voltage, to better highlight the active power changes during the charging of an electric vehicle.

In stage 1 of characteristic in Figure 16, a slow linear increase of average values of active power can be seen. In stage 2a the nature of the charging process changes. Stage 2a starts with a step increase in average values of active power. At the same time, there is an increase in active power changes. In stage 2, there is a trend of increasing active power associated with several step changes in the average values of active power. In stage 2b, there is a reduction in active power changes compared to stage 2a. Short-term undervoltages cause short-term increases in power active changes. However, due to the short duration of this phenomenon, it does not have a significant impact on the charging process. It should be noted that the largest active power changes (Figure 14, Figure 15 and Figure 16) are not correlated with undervoltages.

**Figure 17:**
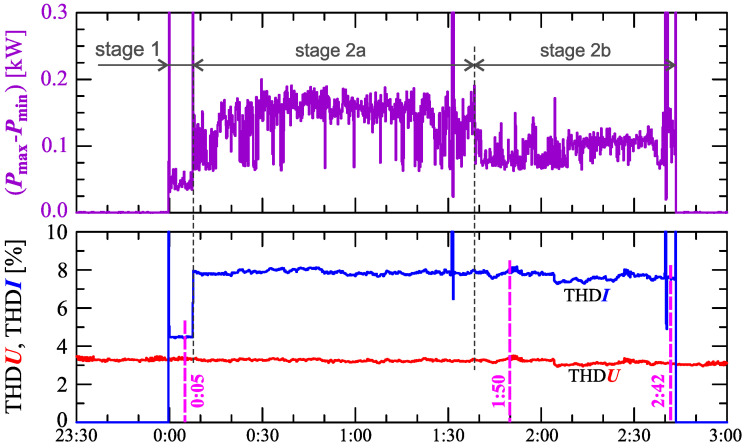
The difference between the maximum and minimum active power $\left(P_{\max}-P_{\min}\right)=f\left(t\right)$ (top), the voltage THD factor THDU=f(t), and current THD factor THDI=f(t) (bottom) with the markings of measurement results presented on the waveforms (Figure 21) and amplitude spectra (Figure 20) recorded at 0:05, 1:50, and 2:42 for Case II.

Figure 17 shows characteristics of the voltage and current THD factors and the characteristics of the second harmonics of the current and voltage. The characteristic in Figure 17 clearly shows the increase in power changes $\left(P_{\max}-P_{\min}\right)$ in successive stages. In stage 1 the variation is approximately 50W, in stage 2a it increases to approximately 160W, in stage 2b it decreases to approximately 100W.

The indicated stages of the charging process are determined by analyzing the nature of active and reactive power. However, as with Case I, stages/sub-stages of the charging process can be determined by analyzing current and voltage harmonics. Figure 17 also shows the characteristics of the current and voltage THD factors. Comparing the characteristics of the current and voltage THD factors, it can be noticed no dependency between changes in the current and voltage distortion (i.e., the increase in the current distortion is not caused by the increase in the voltage distortion). The characteristic of the current THD factor confirms the division of the charging process into two stages. In stage 1, values of the current THD factor are slightly greater than values of the voltage THD factor. In stage 2, values of the current THD factor increase significantly while the voltage THD factor are kept (approximately) constant.

**Figure 18:**
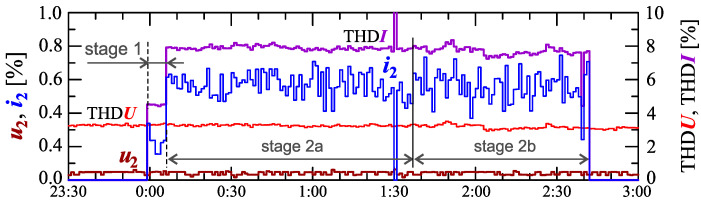
The characteristics of the voltage THD factor THDU=f(t) and current THD factor THDI=f(t) and the characteristics of the second harmonics of the current $i_{2}=f\left(t\right)$ and voltage $u_{2}=f\left(t\right)$ for Case II.

Information about the charging process can be provided by analyzing individual higher harmonics. Considering the properties of the DFT algorithm, even harmonics can be expected in the case of power variation (thus current variations). Figure 18 shows characteristics of the voltage and current THD factors and the characteristics of the second harmonics of the current and voltage.

**Figure 19:**
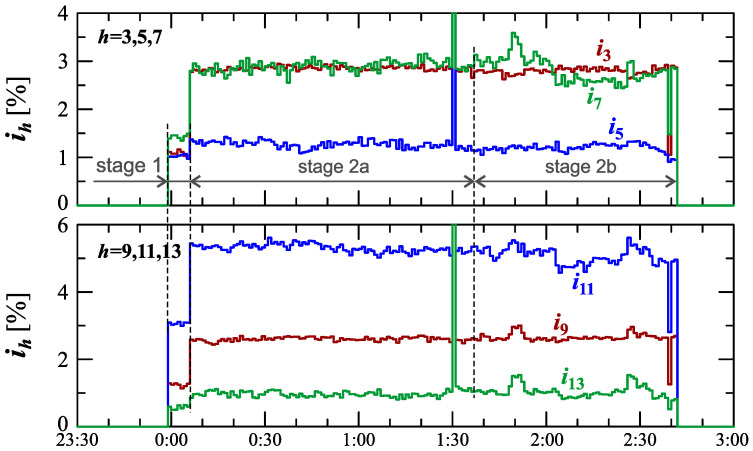
The characteristics of odd harmonics of the current $i_{h}=f\left(t\right)$ from the order *h* = 3 to 13 for Case II.

As expected, the occurrence of the second harmonic of the current is correlated with the occurrence of the current variation. The characteristic of this harmonic suggests, similar to the characteristic of the current THD factor, the division of the charging process into two stages. Comparing Figure 18 with Figure 16 and Figure 17, it can be seen that the increase in active power changes is associated with an increase in the second harmonic of the current. However, based on Figure 17, it is difficult to determine the sub-stages (stage 2a and stage 2b) in stage 2. In order to obtain information about these sub-stages, the occurrence of higher harmonics of the current should be analyzed. The characteristics of odd harmonics of the current $i_{h}$ from the order *h* = 3 to 13 are shown in Figure 19.

Analyzing characteristics $i_{h}=f\left(t\right)$ in Figure 19 for harmonics *h* = 3, 5, 9, 11, and 13, stages 1 and 2 can be easily identified. Determination of sub-stages in stage 2 is possible only on the basis of the nature of the 7th harmonic of the current. In stage 2a, the 3rd and 7th harmonic values are close, and in stage 2b, the 7th harmonic value is noticeably different compared to the 3rd harmonic. The increase in the contribution of the odd harmonics in stage 2 is probably related to the operation of the power electronic converter in the charging system, causing harmonics of the order of 6n±1, where *n* is any natural number. The tendency in the nature of harmonics h>13 is analogous to Case I; therefore, the presentation of characteristics of these harmonics is omitted. Analyzing characteristics of the current THD factor in Figure 17 and Figure 18 and the characteristics of higher harmonics $i_{h}=f\left(t\right)$ in Figure 19, an increase in current distortion can be seen.

**Figure 20:**
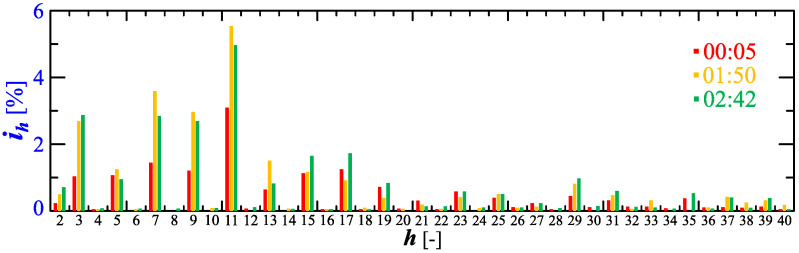
The amplitude spectrum of the current $i_{h}=f\left(h\right)$ recorded at 00:05, 1:50, and 2:42 for Case II.

**Figure 21:**
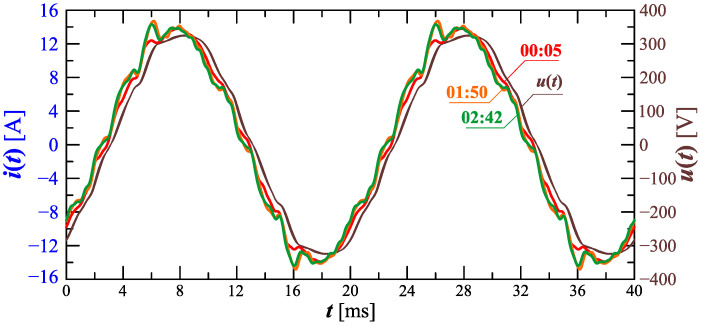
Recreated voltage waveforms u(t) and current waveforms i(t) recorded at 00:05, 1:50, and 2:42 for Case II.

Figure 20 shows the amplitude spectrum of the current recorded at 00:05, 1:50, and 2:42 (recording times are marked in Figure 17). Recreated current waveforms on the basis of the recorded harmonics are shown in Figure 21.

**Figure 22:**
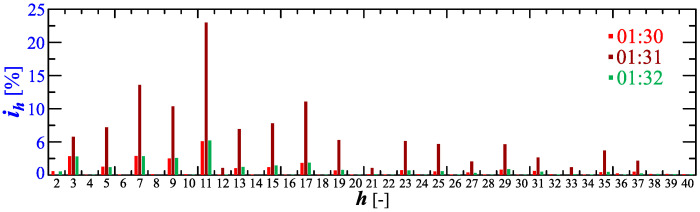
The amplitude spectrum of the current $i_{h}=f\left(h\right)$ recorded at 01:30, 01:31, and 01:32 for Case II.

**Figure 23:**
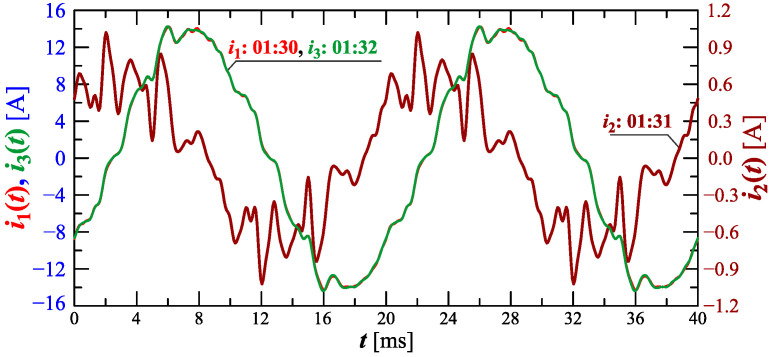
Recreated voltage waveforms u(t) and current waveforms i(t) recorded at 01:30, 01:31, and 01:32 for Case II.

Figure 20, Figure 21, Figure 22 and Figure 23 confirm the non-stationarity of the charging process characterized by the changes in the current waveform and, thus, the changes in the contribution of individual harmonic values in successive time steps. Based on waveforms presented in Figure 21 and Figure 23, conclusions can also be made with regard to the requirements of measuring equipment used to measure selected quantities when charging an electric vehicle. Namely, it is worth noting that the current waveform is strongly non-stationary, and as a consequence, it can lead to significant errors in the measurement of higher harmonics, considering the requirements of the currently used power quality analyzers. Minimizing errors in determining harmonics for waveforms whose nature is similar to the current consumed by the EV onboard charger requires a preliminary algorithm for effective detection of quasi-fundamental periods. Such a procedure also ensures the minimization of errors in determining other parameters for periodic signals.

Figure 22 and Figure 23 show the amplitude spectrum and recreated current waveforms at 1:30, 1:31, and 1:32.

### 3.3. Other Cases

Cases III–V are presented in one subsection because the trends in changes in the rms value, $P_{st}$ indicator, higher harmonics with order greater than the 13th order, or changes in waveforms of the current consumed by the EV onboard charger are similar to Case I and Case II. Characteristics of changes in the active power, reactive power, voltage, and current THD factors, and harmonics up to the 13th order are presented below because they allow indicating a slightly different nature of the phenomenon occurring during the monitored charging process of an electric vehicle.

**Figure 24:**
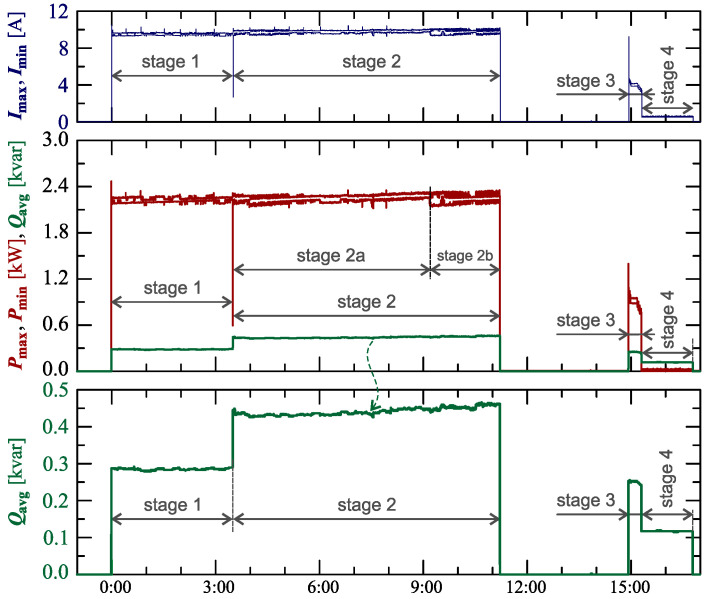
The characteristics of maximum $P_{\max}=f\left(t\right)$ and minimum $P_{\min}=f\left(t\right)$ values of active power and average values of reactive power $Q_{\mathrm{avg}}=f\left(t\right)$ for Case III.

**Figure 25:**
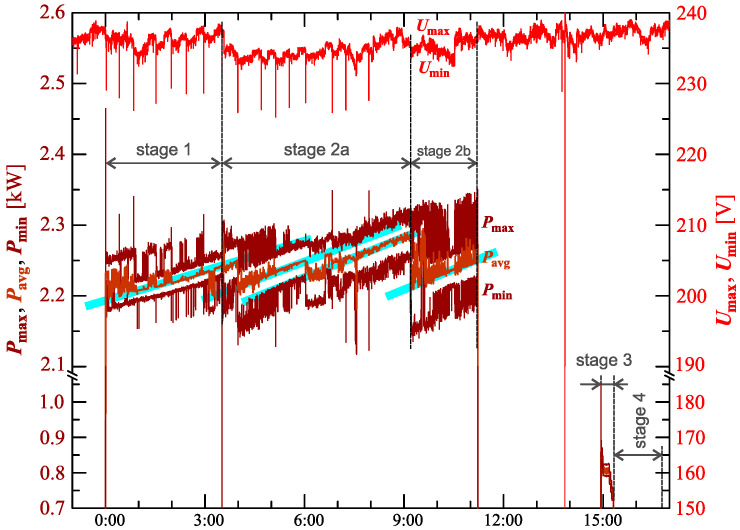
The characteristic of maximum $P_{\max}=f\left(t\right)$, average $P_{\mathrm{avg}}=f\left(t\right)$ and minimum $P_{\min}=f\left(t\right)$ values of active power and maximum $U_{\max}=f\left(t\right)$ and minimum $U_{\min}=f\left(t\right)$ rms values of supply voltage for Case III; cyan lines are added in the background to indicate the quasi-linear increase in average values of active power.

**Figure 26:**
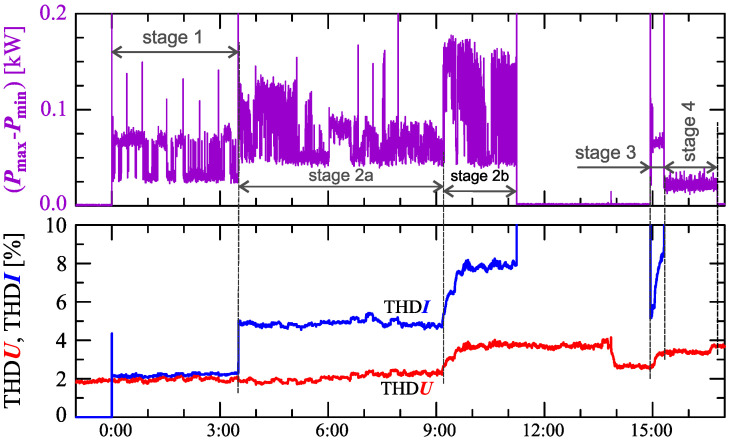
The characteristic of difference between the maximum and minimum active power $\left(P_{\max}-P_{\min}\right)=f\left(t\right)$ (top), and the characteristics of the voltage THD factor THDU=f(t) and current THD factor THDI=f(t) (bottom) for Case III.

**Figure 27:**
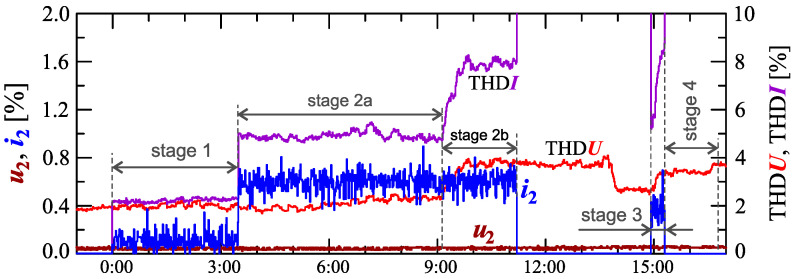
The characteristics of the voltage THD factor THDU=f(t) and current THD factor THDI=f(t) and the characteristics of the second harmonics of the current $i_{2}=f\left(t\right)$ and voltage $u_{2}=f\left(t\right)$ for Case III.

**Figure 28:**
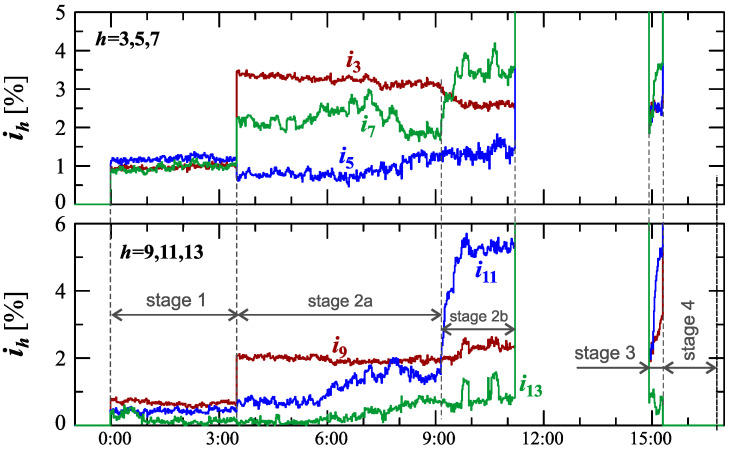
The characteristics of odd harmonics of the current $i_{h}=f\left(t\right)$ from the order *h* = 3 to 13 for Case III.

Figure 24, Figure 25, Figure 26, Figure 27 and Figure 28 show the measurement results during the charging process defined as Case III. Two dominant stages: 1 and 2 (main charging phase) and two additional stages: 3 and 4 (additional charging phase) can be indicated on the basis of changes in reactive power (Figure 24). Two sub-stages (stage 2a and stage 2b) can be determined in stage 2. Figure 24 also shows the characteristics of maximum $I_{\max}=f\left(t\right)$ and minimum $I_{\min}=f\left(t\right)$ values of rms values of the current. Figure 25 shows characteristics of maximum $P_{\max}$, average $P_{\mathrm{avg}}$ and minimum $P_{\min}$ of active power, on a properly scaled characteristic, in relation to the characteristics of maximum $U_{\max}$ and minimum $U_{\min}$ rms values of the voltage, to better highlight active power changes during charging of an electric vehicle. In stages 1, 2a, and 2b, there is a trend of linear increase in the active power consumed with distinct step changes in the active power value. The characteristic of average values of active power has a shape similar to the sawtooth signal. Quasi-linear power increases can be explained by charging the battery pack in constant current mode. The characteristic $\left(P_{\max}-P_{\min}\right)=f\left(t\right)$ in Figure 26 shows that during the tested charging process, the active power often changes. In stage 1, the active power changes are between 25W and 75W. In stage 2a, the active power changes are between 50W and 125W. In stage 2b, the active power changes are over 150W. In stage 3, the active power changes are associated with both stage 1 and stage 2. Comparing the values of active and reactive power (of a capacitive nature) in stage 4 (Figure 24) and active power changes Figure 26 in stage 4, it can be assumed that the charging system in stage 4 is in standby mode (until the charging circuit plug is disconnected). In this mode, there is reactive power associated with the charging of capacitors at the inputs of converter systems. Figure 26 also shows the characteristics of the current and voltage THD factors. Comparing the characteristics of the current and voltage THD factors, unlike Case I and II, it can be seen a certain relationship between the current and voltage distortion in stage 2b of the main charging phase and stage 3 of the additional charging phase—the increase in current distortion is correlated with the increase in voltage distortion. In other stages/sub-stages, there is no relationship between the voltage and current THD values. Considering no dependency between the THD factor of the current and voltage in other stages (including other cases) and the fact that the battery pack charging system is supplied at a close distance to the MV/LV transformer (low impedance of the power supply circuit), it can be concluded that the THD factor of the voltage noticeably affects the THD factor of the current. Thus, the deterioration of the voltage quality amplifies the deterioration of the current consumed by the charger system. The characteristic of the current THD factor in Figure 26 confirms the division of the charging process into stages proposed on the basis of characteristics of active and reactive power (Figure 24 and Figure 25). Figure 27 shows characteristics of the voltage and current THD factors and the characteristics of the second harmonics of the current and voltage. The characteristic of the second harmonic of the current suggests the division of the charging process into three stages, from 1 to 3, omitting stage 4. Analyzing the characteristics of odd harmonics in Figure 28, different characters of individual harmonics than in Cases I and II can be noticed. Each of the higher harmonic characteristics suggests the division of the charging process into three stages (similar to the active power characteristics in Figure 24, Figure 25 and Figure 26). In stage 1, higher harmonic steps change their values to specific levels (with low variations). Between stage 1 and stage 2, there is a next step change in the harmonic values. In stage 2, the characteristic of the 3rd harmonic has a downward tendency, and the characteristics of other harmonics have an upward tendency. The 7th and 11th harmonics indicate the determination of two sub-stages (stage 2a and stage 2b) in stage 2.

**Figure 29:**
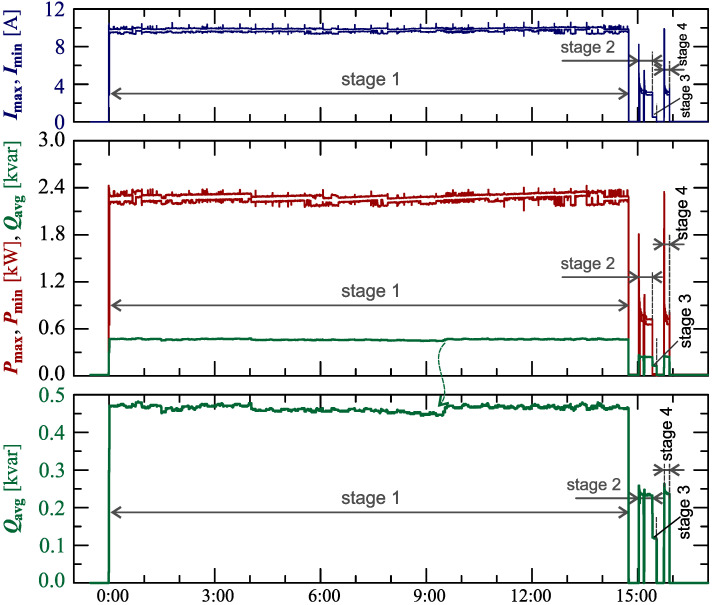
The characteristics of maximum $P_{\max}=f\left(t\right)$ and minimum $P_{\min}=f\left(t\right)$ values of active power and average values of reactive power $Q_{\mathrm{avg}}=f\left(t\right)$ for Case IV.

**Figure 30:**
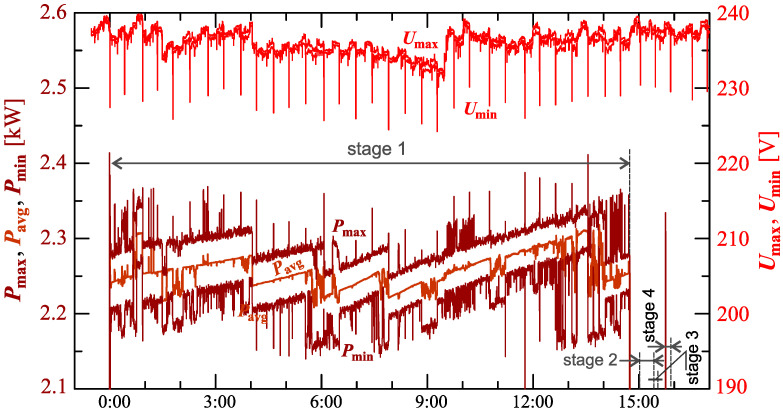
The characteristic of maximum $P_{\max}=f\left(t\right)$, average $P_{\mathrm{avg}}=f\left(t\right)$ and minimum $P_{\min}=f\left(t\right)$ values of active power and maximum $U_{\max}=f\left(t\right)$ and minimum $U_{\min}=f\left(t\right)$ rms values of supply voltage for Case IV.

**Figure 31:**
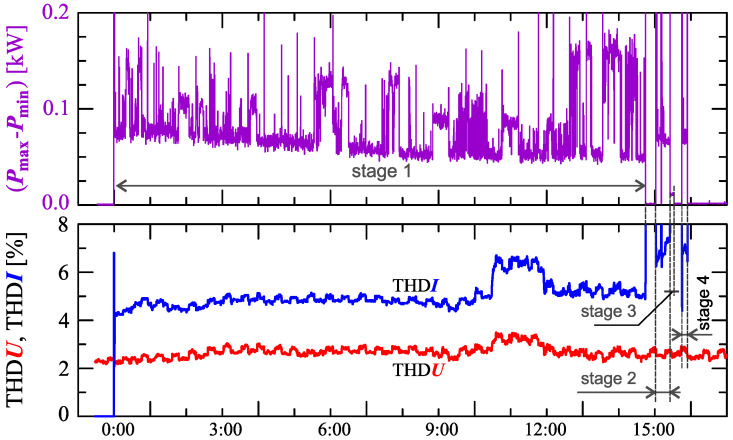
The characteristic of difference between the maximum and minimum active power $\left(P_{\max}-P_{\min}\right)=f\left(t\right)$ (top), and the characteristic of the voltage THD factor THDU=f(t) and current THD factor THDI=f(t) (bottom) for Case IV.

**Figure 32:**
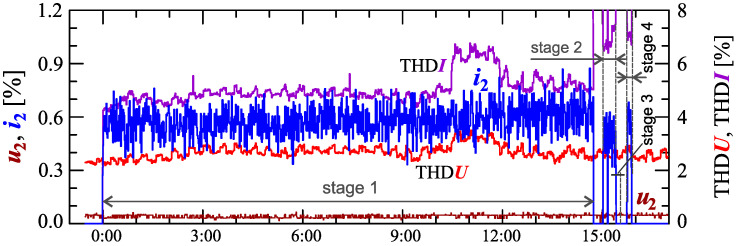
The characteristics of the voltage THD factor THDU=f(t) and current THD factor THDI=f(t) and the characteristics of the second harmonics of the current $i_{2}=f\left(t\right)$ and voltage $u_{2}=f\left(t\right)$ for Case IV.

**Figure 33:**
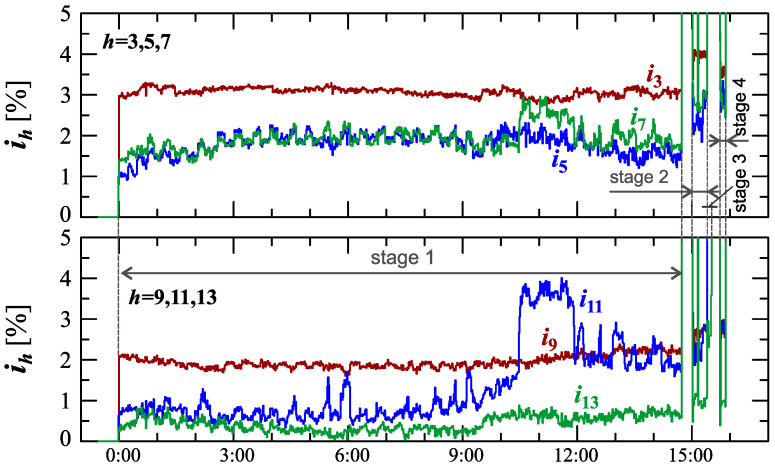
The characteristics of odd harmonics of the current $i_{h}=f\left(t\right)$ from the order *h* = 3 to 13 for Case IV.

Figure 29, Figure 30, Figure 31, Figure 32 and Figure 33 show the measurement results during the charging process defined as Case IV. Characteristics of active and reactive power (Figure 29) show the main charging phase (stage 1), and two additional charging phases (stages 2–4). The first additional charging phase consists of two stages (stages 2–3), and stage 4 is the second additional charging phase. Stage 3 is the standby mode of the charging system (like stage 3 for Case III). Stages 2 and 4 are short-term supplementary charging with active and reactive power changes similar to stage 1 (Figure 31). However, for stages 2 and 4, the current distortion is significantly higher than for stage 1 (Figure 31, Figure 32 and Figure 33). This is especially visible for the 3rd and 5th harmonics. In Figure 30, it can be seen that the increase in values of the voltage THD factor is related to the increase in values of the current THD factor associated with the increase in 11th and 7th harmonics (Figure 31 and Figure 33).

**Figure 34:**
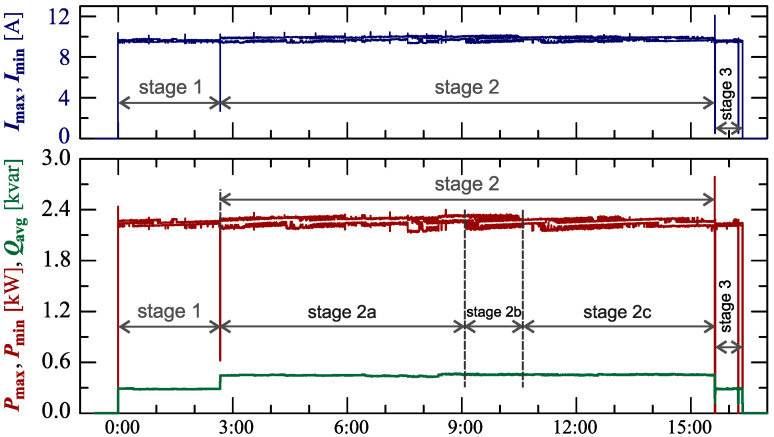
The characteristics of maximum $P_{\max}=f\left(t\right)$ and minimum $P_{\min}=f\left(t\right)$ values of active power and average values of reactive power $Q_{\mathrm{avg}}=f\left(t\right)$ for Case V.

**Figure 35:**
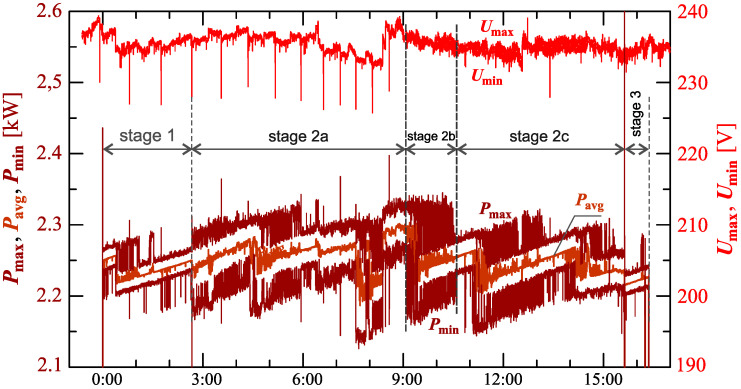
The characteristic of maximum $P_{\max}=f\left(t\right)$, average $P_{\mathrm{avg}}=f\left(t\right)$ and minimum $P_{\min}=f\left(t\right)$ values of active power and maximum $U_{\max}=f\left(t\right)$ and minimum $U_{\min}=f\left(t\right)$ rms values of supply voltage for Case V.

**Figure 36:**
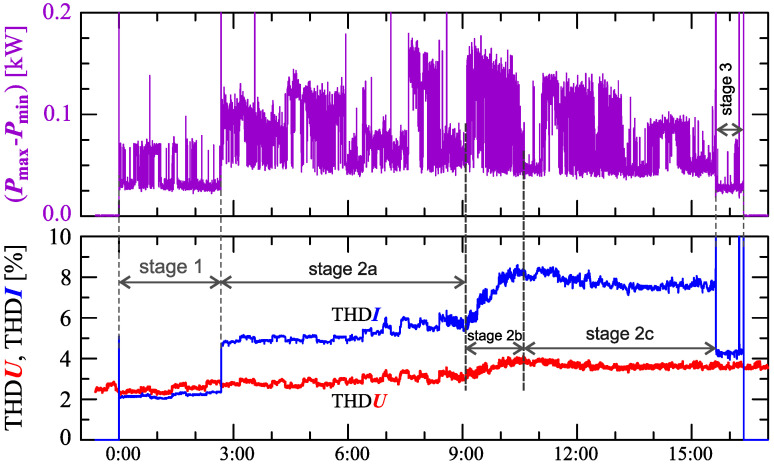
The characteristic of difference between the maximum and minimum active power $\left(P_{\max}-P_{\min}\right)=f\left(t\right)$ (top), and the characteristic of the voltage THD factor THDU=f(t) and current THD factor THDI=f(t) (bottom) for Case V.

**Figure 37:**
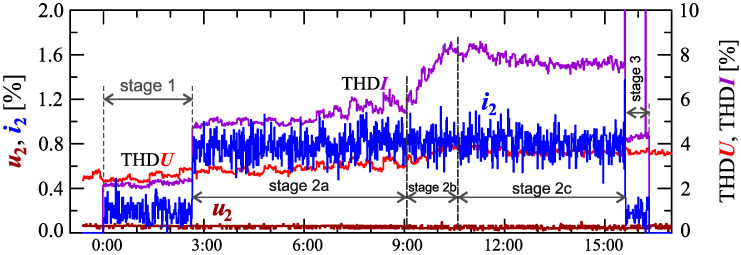
The characteristics of the voltage THD factor THDU=f(t) and current THD factor THDI=f(t) and the characteristics of the second harmonics of the current $i_{2}=f\left(t\right)$ and voltage $u_{2}=f\left(t\right)$ for Case V.

**Figure 38:**
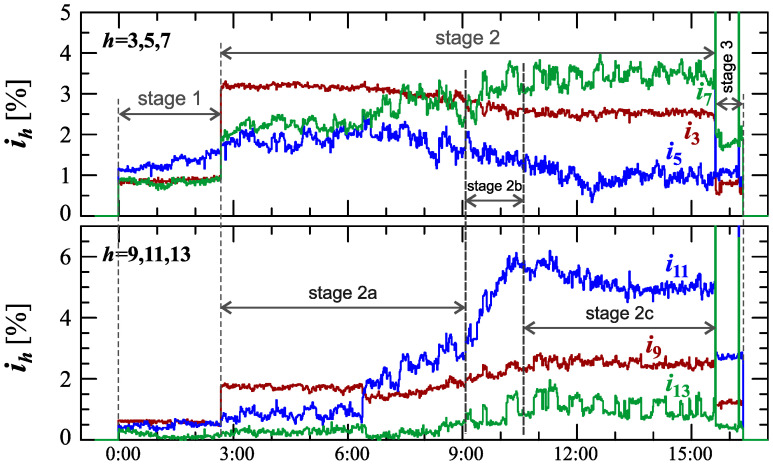
The characteristics of odd harmonics of the current $i_{h}=f\left(t\right)$ from the order *h* = 3 to 13 for Case V.

Figure 34, Figure 35, Figure 36, Figure 37 and Figure 38 show results of measurements for the longest charging process among those presented, defined as Case V. The characteristic of reactive power in Figure 34 shows that the charging process consists of 3 stages. Based on Figure 34 and Figure 35, it can be concluded that the smallest active power changes occur in stages 1 and 3. Analyzing characteristics of active power, stage 2 can be divided into different sub-stages. For the purpose of the charging process analysis, a division into 3 sub-stages: 2a, 2b, and 2c, is adopted. Stage 2b has the highest changes in the active power. Stages 2a and 2c have comparable active power changes. In stage 2c there is a greater current distortion than in stage 2a. The increase in the value of the THD factor in stage 2c (Figure 38) results from the increase in harmonics from *h* = 7 to 13 with a simultaneous decrease in the 3rd and 5th harmonics. It is worth noting that stages 1 and 3, despite the similarity of characteristics of active and reactive power, stages differ in current distortion. This can be due to the increase in the value of the voltage THD factor in stage 3 relative to stage 1, with the influence of the THD values on the individual harmonics of the current being different.

**Figure 39:**
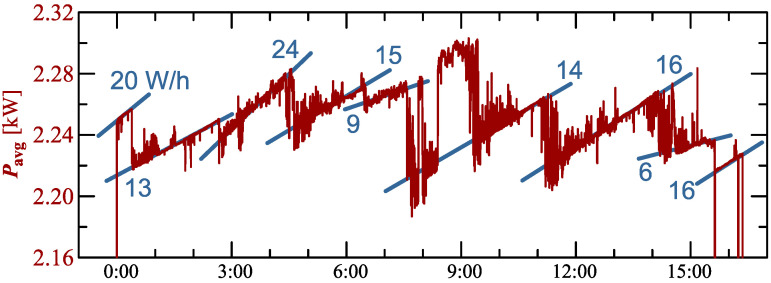
The characteristic of the average values of active power for Case V with information about the slope in graphical (trend lines) and numerical (value in W/h) form.

In the presented charging processes (Figure 5, Figure 15, Figure 25, Figure 30 and Figure 35) characteristic of average values of active power is similar to the sawtooth signal. Analyzing indicated characteristics, it can be seen that the slope of individual teeth of the sawtooth signal is different. Figure 39 shows the characteristic of average values of active power for Case V with information about the slope in graphical (trend lines) and numerical (value in W/h) form.

The characteristic of average values of active power indicates that charging is a complex and non-monotonic process. This allows for the conclusion that the charging process is characterized by a high variation of average and instantaneous values of active power.

**Figure 40:**
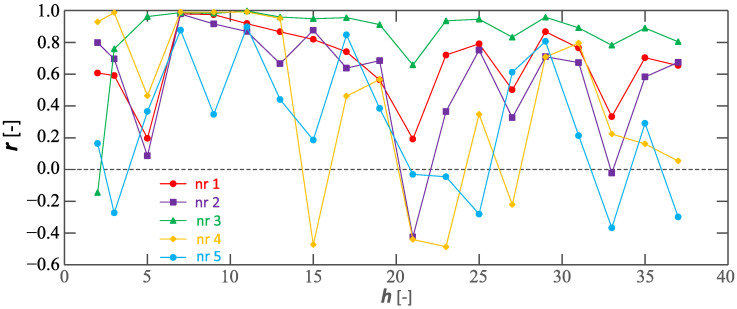
Correlation *r* coefficient of characteristics of *h*-th harmonics of the voltage and current for considered charging processes, which are defined as Cases I–V.

Analyzing the charging processes referred to as Cases I–V, it is worth noting the relationship between the individual voltage and current harmonics. Figure 40 shows the calculated correlation *r* values of the *h*–th harmonics of the voltage and current for Cases I–V. Pearson’s linear correlation *r* coefficient is calculated according to the relation:

(1) $$r=\frac{\sum_{i=1}^{N}\left(x_{i}-\bar{x}\right)\left(y_{i}-\bar{y}\right)}{\sqrt{\sum_{i=1}^{N}{\left(x_{i}-\bar{x}\right)}^{2}}\sqrt{\sum_{i=1}^{N}{\left(y_{i}-\bar{y}\right)}^{2}}},$$

where *x* and *y* are the considered measurement series included in the statistical analysis (e.g., selected *h*-th harmonic of the voltage and current over time), $\bar{x}$ and $\bar{y}$ are the calculated values of the arithmetic mean for the series marked as *x* and *y*, respectively.

Analyzing characteristics shown in Figure 40, it can be seen that for some voltage and current harmonics, in particular for *h* = 7, 11, and 29, a strong statistical relationship is visible. When considering the relationships between individual harmonics, it is worth noting that the EV onboard charger is supplied by a low-impedance line. In this case, the effect of the current distortion should not significantly affect the voltage distortion, i.e., a strong statistical relationship between voltage and current is not expected. This is consistent with the observations of Cases I–V. The cases in which there is a strong statistical relationship for specific harmonics are difficult to explain. Perhaps this is the result of the occurrence of states close to parallel resonance, for which small values of the current can cause significant values of the voltage. Detection of such states is problematic, especially since the properties of the power supply circuit can change in real-time (connection and disconnection of different loads, including those of a resistive–capacitive nature).

The results of monitoring the selected cases presented in the paper show the non-stationarity of the phenomena occurring during the charging of an electric vehicle. The onboard charger is a power electronics system. Therefore, it is difficult to determine the impact of such a system on power quality during charging. This results in the postulate of supervision of the charging process, especially at the beginning of EV use. It should be emphasized that the effects of EV onboard charger operation can be amplified with an increasing impedance of the power supply circuit. Determination of the impact of charging on power quality requires considering the possible operation of other EV onboard chargers, e.g., at a neighbor connected to the same LV line.

## 4. Conclusions

The paper presents the results of the monitoring of the process of charging an electric vehicle battery pack in a single-phase 230V/50Hz circuit. This process is managed by the EV onboard charger. In the monitoring process, the focus is on the recognition of the charging process, considering the impact of this process on the power quality and consequently on the reliability of electric machines. The research results indicate that the monitored processes of the charging of an electric vehicle are non-stationary, and are characterized, for example, by different numbers of stages/sub-stages, within which individual parameters assume specific values. The individual stages/sub-stages are characterized by variations of the currents, active and reactive power, and higher harmonic contributions. The observed changes have different causes. The quasi-linear slow increase of active power is related to the increase of the voltage on the battery pack during charging. Other types of changes, which have short-term phenomena, are associated with the operation of an EV onboard charger. In particular, the operation of the PFC system can cause changes in the current and power. The operation of the EV onboard charger depends on the control algorithm implemented in it. If EV onboard chargers have compatible topologies, it can be expected that the variation of powers (active and reactive) and parameters assessing power quality can be similar. However, for different topologies, it is advisable to carry out the research presented in the paper. The effects of current, active, and reactive power and higher harmonics changes depend on the characteristics of the power grid at the point of connection of the charging system. The effects of such changes can be amplified if the vehicle’s charging system is supplied by a high-impedance line. Knowledge of the voltage, current, and power variation allows for the specification of requirements for the measurement equipment used to monitor the charging process, including the determination of the discrimination/averaging time of the monitored quantities. The obtained research results indicate the need for continuous monitoring of power quality in the particular circuit, where electrical loads (e.g., electric machines) are supplied. Continuous monitoring supports the diagnosis of electrical machines and allows for appropriate actions to increase their reliability.

## Figures and Tables

**Table 1:** Summary of the selected charging processes.

Case No.	$E_{P}$ [kWh]	$E_{Q}$ [kvarh]	$T_{\mathrm{charge}}$ [min]	$T_{\mathrm{REC}}$ [s]	Remarks
I	3.9	0.6 lead	104	30	Main charging phase consisting of 2 stages (2 sub-stages in stage 2)
II	6.1	1.2 lead	164	10	Main charging phase consisting of 2 stages (2 sub-stages in stage 2)
III	25.5	4.7 lead	674	10	Main charging phase consisting of 2 stages (2 sub-stages in stage 2) and additional charging phase consisting of 2 stages
IV	33.7	6.8 lead	884	20	Main charging phase consisting of one stage and two additional phases consisting of two and one stage respectively
V	36.7	6.8 lead	982	10	Main charging phase consisting of 3 stages (3 sub-stages in stage 2)

## Data Availability

Not applicable.
